# Mediterranean diet and Cantonese cuisine for human health: report from a Sino-Italian bilateral meeting

**DOI:** 10.1007/s40520-025-03199-x

**Published:** 2025-10-11

**Authors:** Stefania Maggi, Fiona Ecarnot, Vincenza Gianfredi, Daniele Nucci, Nicola Veronese, Liang Lei, Min Hu, Christelle Avart, Antonio Capurso, Limin Chen, Fatima Hachem, Haibin Yang, Antonio F. Logrieco, Massimiliano Magli, Quansheng Mai, Federico Palla, Stefano Predieri, René Rizzoli, Domenico Rogoli, Angelo Santino, Marco Silano, Milena Simeoni, Antonia Trichopoulou, Roberto Volpe, Yong Wang, Juhua Wu, Ma Xiaohui, Xiaoyan Chen, Xuhui Zhang, Li Yujie, Michela Zanetti, Giorgio Picci

**Affiliations:** 1 National Research Council, Neuroscience Institute, Aging Branch, Padova, Italy; 2 Cardiology Department, University Hospital Besancon, 25000 Besancon, France; 3 SINERGIES laboratory, University Marie & Louis Pasteur, 25000 Besancon, France; 4 Department of Cardiac Thoracic Vascular Sciences and Public Health, University of Padua, 35128 Padova, Veneto, Italy; 5 Department of Public Health, Experimental and Forensic Medicine, University of Pavia, 27100 Pavia, Italy; 6 Struttura Semplice Dipartimentale Igiene Alimenti E Nutrizione, Dipartimento Di Igiene E Prevenzione Sanitaria, Azienda Di Tutela Della Salute (ATS) Brescia, Via Duca Degli Abruzzi, 15, 25124 Brescia, Italy; 7 Faculty of Medicine, Saint Camillus International University of Health Sciences, Rome, Italy; 8 Guangdong Engineering Research Center of Chinese Medicine & Disease Susceptibility, College of Traditional Chinese Medicine, Jinan University, Guangzhou, 510632 China; 9 Department of Traditional Chinese Medicine, The First Affiliated Hospital of Guangzhou Medical University, Guangzhou, 510000 China; 10 Institute of Integrated Traditional Chinese Medicine and Western Medicine, Guangzhou Medical University, Guangzhou, 510000 China; 11 Nutrition, Scientific affairs and regulatory manager at CPW – Cereal partners worldwide – JV Nestlé and General Mills, Chair of Food policy working group of Whole Grain Initiative, Vienna, Austria; 12 Department of Internal Medicine and Geriatrics, School of Medicine, University of Bari Aldo Moro, Bari, Italy; 13 Tea Art Instructor of the Guangzhou Vocational School of Tourism and Business, Guangzhou, China; 14 Senior Nutrition Officer Nutrition Education and Consumer Awareness Team Leader, Food and Nutrition Division, FAO, Rome, Italy; 15 Chinese Director of the Confucius Institute at the University of Padova, Padova, Italy; 16 Xianghu Lab, Biomanufacturing Institute, Hangzhou, Zhejiang Province China; 17 Institute for BioEconomy, National Research Council of Italy (CNR), Bologna, Italy; 18 Office of International Exchanges and Cooperation Guangzhou, Vocational School of Tourism and Business, Guangzhou, China; 19 Fondazione Internazionale LUMEN ETS, Piacenza, Italy; 20 Institute for BioEconomy, National Research Council of Italy (CNR), Bologna, Italy; 21 Faculty of Medicine, Geneva University Hospitals, Geneva, Switzerland; 22 Institute of Sciences of Food Production, CNR, Unit of Lecce, Lecce, Italy; 23 Department of Cardiovascular, Endocrine-metabolic Diseases and Aging, Istituto Superiore di Sanità, Rome, Italy; 24 Adjunct Professor Yale School of Public Health, New Haven, CT USA; 25 Prevention Unit, National Research Council of Italy (CNR), Rome, Italy; 26 Guangzhou Vocational School of Tourism and Business, Guangzhou, China; 27 Chinese Culinary Instructor of the Guangzhou Vocational School of Tourism and Business, Guangzhou, China; 28 Confucius Institute, University of Venice, Venice, Italy; 29 Department of Endocrinology, The First Affiliated Hospital of Guangzhou Medical University, Guangzhou, 510000 China; 30 Department of Oncology, Guangdong Second Provincial General Hospital, The Affiliated Guangdong Second Provincial General Hospital of Jinan University, Guangzhou, China; 31 The Second School of Clinical Medicine of Southern Medical University, Guangzhou, 510317 China; 32 Chinese Teacher of the Confucius Institute at the University of Padova, Padova, Italy; 33 Depatment of Medical, Surgical and Health Sciences, UCO Geriatria, University of Trieste, Trieste, Italy; 34 Director of the Confucius Institute at the University of Padova, Padova, Italy

**Keywords:** Mediterranean diet, Cantonese cuisine, Cardiovascular disease, Cancer prevention, Functional foods, Nutrition, Public health, Traditional medicine, Dietary patterns, Sustainability

## Abstract

This article explores the traditional Mediterranean and Cantonese diets through historical, cultural, and scientific lenses. Drawing from expert presentations delivered during a multi-day international symposium, we examine the culinary practices, nutritional components, and health implications of both dietary traditions. The comparative analysis addresses cardiovascular and metabolic health, cancer prevention, functional foods, public policy, and the emerging role of traditional foods in modern preventive medicine. By analyzing the synergy between dietary elements and lifestyle factors, we highlight how these long-standing traditions can inform contemporary strategies for health promotion and chronic disease prevention.

## Introduction

There is increasing recognition that dietary patterns are major determinants of health at an individual level, but also as components of overall public health and sustainable development policies. With current concerns about changing climate conditions, and the inexorable increase in non-communicable diseases, traditional dietary patterns, such as the Mediterranean Diet (MedD) and Cantonese cuisine, offer compelling frameworks rooted in centuries of empirical knowledge. Both of these dietary traditions reflect strong cultural identity, geographical biodiversity, and environmentally sustainable practices.

Against this background, the Mediterranean Diet Foundation, together with the Confucius Institute of the University of Padua, Italy, perceived a need to bring together experts on the Mediterranean and Cantonese dietary patterns, to review the beneficial effects of both on human Health, and brainstorm on Future initiatives that could be implemented to jointly promote these dietary patterns on a wider scale. To this end, a four-day meeting was convened at the Headquarters of the Mediterranean Diet Foundation in Brindisi, Italy, from 11 to 15th May 2025. Speakers were tasked with reviewing the historical perspectives, the key components and their health benefits, while, on the last day of the meeting, stakeholders from governmental bodies, World Health Organization, professional societies, research and educational institutions proposed ideas for initiatives to promote healthy eating patterns across the life course. Here, we summarize the findings from the meeting, and its examination of these dietary systems and their implications for contemporary health promotion.

## Historical and cultural perspectives

The Mediterranean Diet is a very ancient, healthy dietary pattern, based largely on plant-based products, and olive oil as the main source of fat [1, 2]. This diet was originally described in the countries around the Mediterranean basin where the olive tree grows spontaneously. It originated in the Fertile Crescent (covering Mesopotamia and the Nile), and progressed westward through Greece to reach Rome [3]. In ancient Rome, there were two distinct models of diet. The aristocrats ate meat and fish, while the common people, the plebs, ate a mainly vegetarian diet based on a triad comprising bread, olive oil and wine, with, in addition, some legumes and cheeses. Over the centuries, major historical events changed this traditional model. In particular, in the years 568 to 774 AD, the arrival of the “Barbarians” from the north ushered in a tremendous clash between the Greco-Roman and Barbarian civilizations, which were very different from, and alien to each other. Indeed, while the Romans were a stable population with an essentially cereal-based diet, the “Barbarians”, i.e. Celtic and Germanic populations, had, on the contrary, a semi-nomadic lifestyle. They had a silvo-pastoral economy based essentially on the exploitation of woodland, fishing, farming and hunting, such as wild boar, deer and other game. They ate almost exclusively meat, and in this context, cereal-growing had a marginal role since, far from being used to make bread, the grain was mainly used for brewing beer. Over time, however, the two opposing dietary models ended up merging with each other, with each taking on the best features of the other. Accordingly, the barbarian dietary model spread towards to the south, particularly to the inner regions of the Appennine Chain. The very conservative southern population (who had always neglected the wild lands) learned from the barbarians how to exploit these wild territories (by hunting, fishing and collecting wild fruits), and they also learned how to cultivate fresh vegetables. These activities progressively enriched their very frugal diet [3].

There later followed another significant historical event that contributed to evolving the MedD. In the years 827 to 1061 AD, the Arabs conquered Sicily and part of southern Italy. The Arabs brought with them their literary, mathematical, astronomical and geographical culture, and also many new ingredients in the diet, making the Mediterranean diet richer in taste and variety. With the arrival of the Arabs, the focus of the Mediterranean diet shifted from meat towards carbohydrates (namely, dried pasta, rice and sugar), and they also brought numerous new spices, such as cinnamon, ginger, or cloves [3].

The final major historical event to shape the Mediterranean diet was the discovery of the “New World”. In 1492, Christopher Columbus discovered the Americas, and from his four voyages between 1492 and 1502, he brought back many new products to Europe, such as potatoes, pumpkin, beans, maize, tomatoes, sunflowers, avocado, and chocolate. The arrival of these new products were fundamental to feed the European populations of that time, exhausted by wars, plagues and famines. They could finally satisfy their chronic hunger with potatoes, beans, corn, and many new fruit and vegetables. With these last arrivals, the Mediterranean diet was complete, such as it is today. These contributions illustrate the Mediterranean region as a confluence of cultures that shaped a cohesive yet adaptable dietary system.

The history of Cantonese cuisine, a cornerstone of Southern Chinese gastronomy, reflects a similarly dynamic interplay between geography, trade, and cultural integration [4, 5]. It encompasses a wide range of sub-cuisines, including Guangzhou, Teochew, and Hakka, each influenced by terrain, climate, and access to trade routes. The Pearl River Delta, South China Sea, and mountainous northern Guangdong region contribute a diverse array of ingredients: freshwater fish, seafood, tropical fruits, poultry, and wild herbs. The culinary evolution of Cantonese cuisine was also shaped by openness to global influences and the application of sophisticated cooking techniques such as double boiling, claypot cooking, and oil-poaching. The development of Cantonese cuisine (粤菜, Yuè cài) is profoundly rooted in the unique geography, climate, and agricultural systems of the Pearl River Delta in Southern China. Its defining characteristics, namely an emphasis on extreme freshness, mild and sweet flavors, and a plethora of seafood and vegetables, are a direct adaptation to its environment. Cantonese cuisine is not merely a random collection of recipes; it is a logical and delicious adaptation to the abundant freshwater and marine resources, the year-round growing season, and the humid climate of Southern China. The environment dictated the ingredients, and the ingredients dictated the cooking philosophy: pristine freshness above all else [6–9].

Cantonese cuisine is characterized by five tastes (“Gan” or savory-sweet aroma, crispy, tender, fatty and rich) and six flavours (sweet, sour, bitter, spicy, salty and umami). Seasoning is tailored to the ingredients, and Cantonese cooking is famous for this, balancing seasoning and flavours according to the natural taste of the ingredient, to enhance the natural flavour. Cantonese cuisine also relies heavily on sauces to balance the intensity of tastes, with seasoning adjusted according to the seasons (lighter in summer, heavier in winter). A further characteristic of traditional Cantonese cuisine is the pursuit of freshness and delicacy. Strong government support for restaurants and chefs seeking to capitalize on the abundant natural resources, paired with growing demand, have fostered the long-term development of this unique culinary experience.

While geographically and culturally distinct, Cantonese cuisine and the Mediterranean dietary pattern share a fundamental commonality: they are both quintessential expressions of their local environments, shaped by a confluence of geography, climate, and agricultural systems that have dictated their available food sources and culinary philosophies. Similar to Mediterranean Diet, the Cantonese diet is a product of its subtropical monsoon climate, fertile river deltas, and extensive coastline, leading to a cuisine abundant in leafy greens, freshwater and marine seafood, herbs, and tea.

**Fig. 1:**
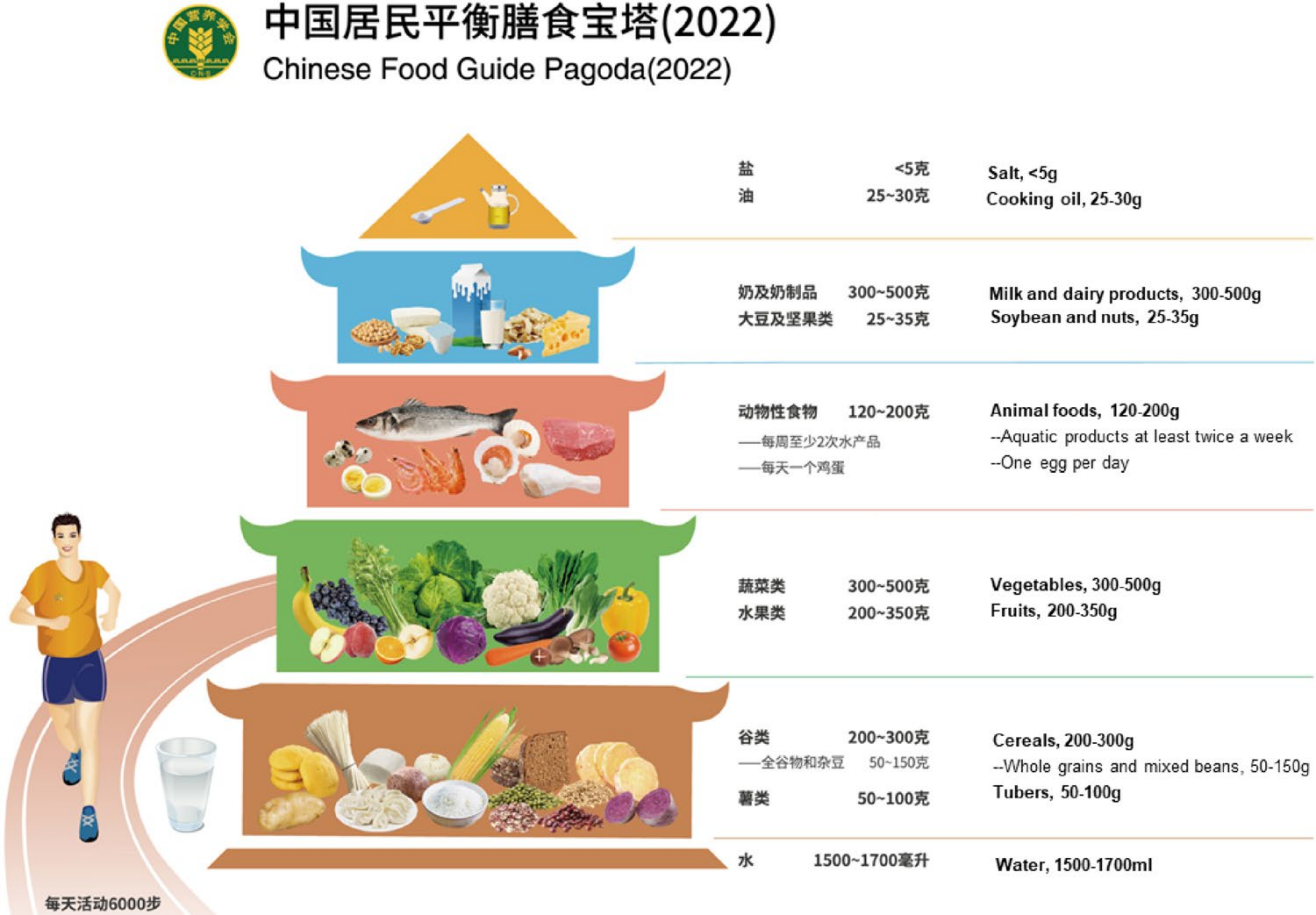
Key components of the Chinese Food Pagoda, with the English translation Available at :https://en.chinacdc.cn/health_topics/nutrition_health/202206/t20220622_259773.html [Access date: 3 September 2025]

**Fig. 2:**
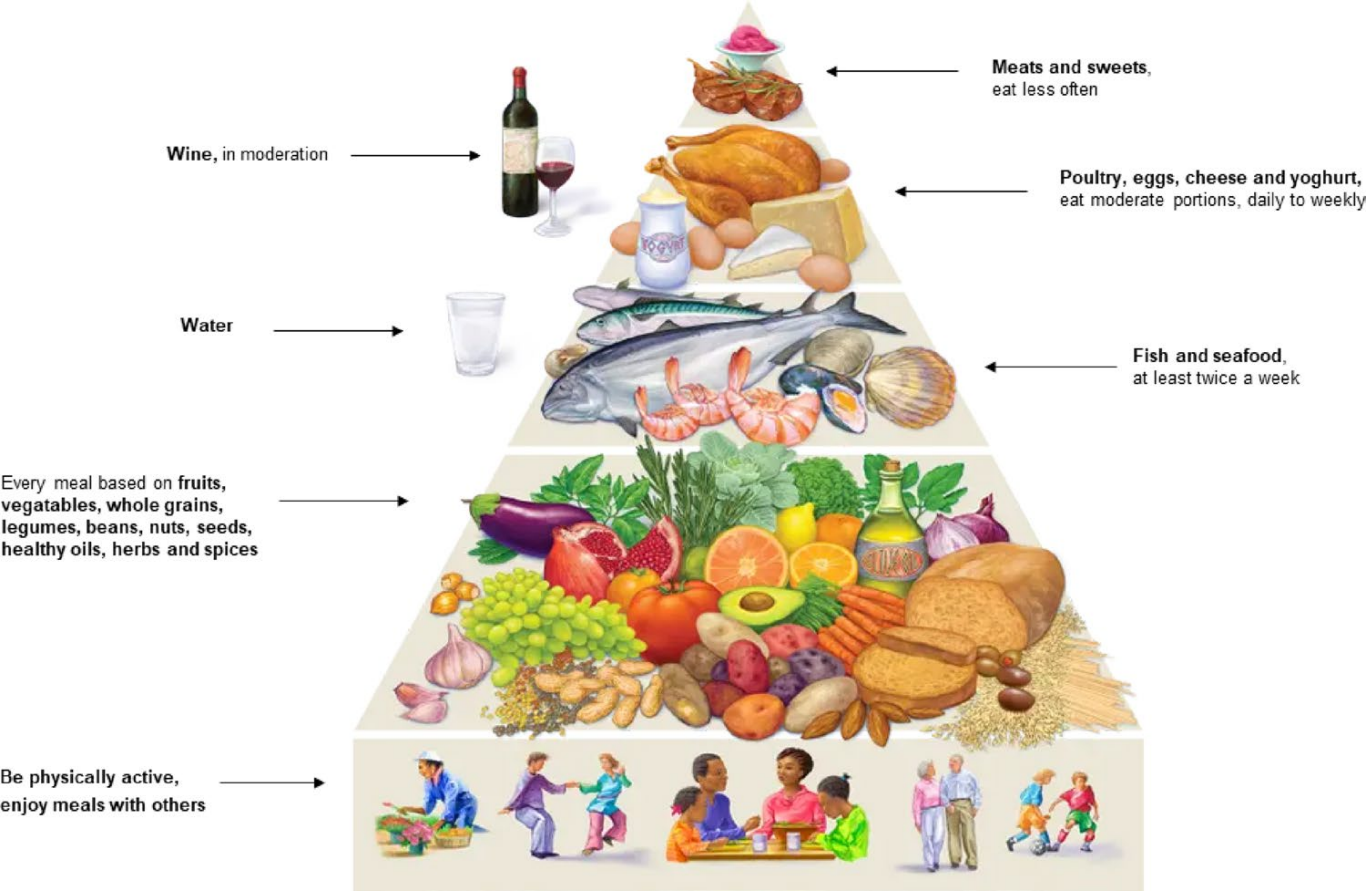
Key components of the Mediterranean Diet Reproduced with permission from Oldways, www.oldwayspt.org

The Chinese food pagoda and the Mediterranean diet pyramid summarize the key dietary recommendations for each dietary pattern (Figs. 1 and 2 respectively).

Cantonese cuisine is an umbrella term encompassing several distinct culinary streams. Table 1 provides a detailed breakdown of its primary categories, to clarify the context for any comparison. While association of Cantonese cuisine with healthy, it is crucial to specify that the potential protective effect is almost certainly linked to the principles of traditional home cooking—high vegetable intake, seafood, steaming, and boiling. This benefit is offset by specific risk factors inherent in other, equally authentic parts of the cuisine: preserved foods (salted fish), high-heat roasting of meats (síu laahm), and extreme wok-frying (street food). Therefore, the overall health outcome for a population depends on the relative consumption frequency of these different sub-categories.

**Table 1:** Specific types of Cantonese cuisine

Category	Primary cooking methods	Key characteristics
Home Cooking	Steaming, Poaching, Light Stir-frying	Focus on fresh ingredients, mild flavors, nutritional balance
Síu Laahm	High-Temperature Roasting/Charcoal Grilling	Caramelized, glazed, and charred meats; rich flavors
Dím Sáam	Steaming, Boiling, Deep-Frying, Pan-Frying	Extreme variety; from delicate steamed dumplings to rich fried snacks
Street Food	Extreme High-Heat Stir-frying (Wok Hei)	Intense, smoky flavors; quick cooking; hearty dishes

## Health benefits of the mediterranean and Cantonese dietary patterns

### Cardiovascular and metabolic health

Dietary patterns play a pivotal role the prevention of cardiovascular disease (CVD). While non-modifiable factors such as sex, aging and genes contribute to disease risk, modifiable Lifestyle factors, especially diet, substantially affect morbidity and mortality. Data from the Global Burden of Disease study 2017 showed that among all major risk factors, dietary factors made the largest percentage contribution to ischaemic Heart disease in terms of age-standardized deaths by sex in 2017 (68.1% in women and 69.7% in men) [10]. Diet contributes to the pathogenesis of CVD both directly and indirectly. In fact, dietary risk is the leading risk in the pathogenesis of CVD because it is linked in turn to atherosclerosis, hypertension, diabetes and obesity, all of which are themselves risk factors for CVD. However, while diets are often thought of in terms of excessive consumption of certain elements (e.g. too much fat or salt), the potential harm associated with the suboptimal consumption of important nutrients is often overlooked, e.g. too Little omega 3, too few grains or vegetables [11]. Accordingly, in a meta-analysis comprising over 5.7 million individuals, higher consumption of ultra-processed foods was shown to be associated with an increased risk of all-cause mortality, while whole-grain cereals were associated with a lower risk of death [12, 13].

The Mediterranean diet has been extensively validated in both observational and interventional studies, including the Seven Countries Study [14] and PREDIMED trial [15], showing that higher adherence to the Mediterranean diet is significantly associated with reductions in hypertension, dyslipidemia, insulin resistance, and overall cardiovascular risk. A meta-analysis totaling 514,816 subjects and 33,576 deaths from eight prospective, observational cohorts confirmed that greater adherence to the Mediterranean diet was associated with a significant reduction in overall mortality (9%), as well as mortality from various chronic diseases, including cardiovascular disease, cancer, and reductions in the incidence of Parkinson’s and Alzheimer’s disease [16, 17]. These findings were corroborated in a more recent umbrella review of meta-analyses [18]. This abundant evidence highlights the relevance of the Mediterranean diet for public health, as an example of good nutrition with the potential to prevent or mitigate various chronic diseases. Consequently, the Mediterranean diet recommended by the European Atherosclerosis/European Society of Cardiology guidelines for the management of dyslipidemias [19], and the European Society of Cardiology guidelines for cardiovascular prevention [20], thanks to the positive health benefits of functional Mediterranean foods, such as fibers (whole cereals, legumes, vegetables, fruit), fish, nuts, and extra-virgin olive oil.

The benefits of the Mediterranean diet on cardiovascular health are mediated by multiple mechanisms. Polyphenols in extra virgin olive oil and fruit activate Nrf2-mediated antioxidant pathways, while omega-3 fatty acids from fish improve lipid profiles and reduce inflammation [21, 22]. Whole grains provide fiber and phytosterols that reduce LDL cholesterol and modulate glycemic response, while fiber-rich foods also support microbiota diversity, which is increasingly linked to cardiometabolic health [22, 23]. In addition, when combined with physical activity, the MedD has been shown to exert positive health benefits on such outcomes as sarcopenia, as well as overall musculoskeletal and cardiorespiratory fitness [24–26].

Data from China show that the Chinese population is similar afflicted by cardiovascular and metabolic diseases, with around one in four Chinese individuals suffering from at least one disease among type 2 diabetes, hypertension, obesity and dyslipidemia [27]. The different eating habits pursued by populations in various geographical regions of China affect overall Health, as shown by a study from over 212 million individuals reporting correlations between different traditional Chinese foods and diabetes risk [28]. The Chinese Dietary Guidelines, released in 2022, were the first to recommend an “Eastern Healthy Diet Model”, underlining that China has developed a healthy dietary model closer to its own eating habits [29]. The background for this model relies on several studies showing low prevalence of overweight/obesity in coastal Chinese areas, and Lower rates of cardiovascular and chronic disease, paralleled by a longer Lifespan. Thus, based on the dietary characteristics of these regions, China proposed a Chinese-specific Healthy diet model, containing 6 core principles (plant-based foods as the mainstay; moderate intake of fish, poultry and eggs; use of plant oils for cooking; use methods such as steaming, boiling or stewing; control salt and sugar intake; take regular and portion-controlled meals). The traditional Cantonese cuisine aligns with these principles for a balanced diet. Indeed, it is rich in dietary fiber, vitamins and minerals, which helps improve gut function, slows down the absorption of blood glucose and blood lipids. It is rich in seafood, a source of high-quality protein and unsaturated fatty acids, while the cooking methods, using primarily steaming and quick stir-frying, help retain nutrients while reducing fat and calorie intake. Modifying traditional recipes through healthier cooking methods, food pairing, and seasoning adjustments can align traditional diets with contemporary health goals.

### Cancer prevention

Adherence to the MedD has been consistently associated with a reduced risk of various types of cancer. This dietary pattern provides a synergistic combination of nutrients and bioactive compounds with anti-inflammatory and antioxidant properties. The Italian national institute for health recently published new national guidelines for the application of the Mediterranean diet in daily life [30]. In this document, an extensive literature review was performed to identify all relevant studies, including randomized controlled trials (RCTs), pre-post intervention studies, and various observational studies (cohort, retrospective, case-control, and cross-sectional) that evaluated the effectiveness of the Mediterranean Diet on several Health outcomes, including cancer risk in the general population. After reviewing 8,314 studies, 126 observational and intervention studies were selected for analysis. Overall, protective effects were noted for head and neck, oral cavity, lung, stomach, liver, colorectal, bladder and breast cancers. The overall effects of the Mediterranean diet on the relative risk for cancer are summarized in Table 2. The observed benefits arise from the MedD’s anti-inflammatory and antioxidant mechanisms, including high intakes of carotenoids, flavonoids, and fiber. Epigenetic modulation and microbiota-mediated immune regulation are emerging as critical pathways.

**Table 2:** Impact of adherence to the mediterranean diet on various types of cancer

Cancer site	Studies	Participants	Relative Risk (95% CI)
Head & neck	3	498,365	0.88 (0.78–0.98)
Oral cavity	5	9555	0.83 (0.73–0.95)
Lung	7	487,206	0.96 (0.94–0.98)
Esophagus	3	619,016	0.94 (0.88–1.002)
Stomach	8	892,261	0.93 (0.88–0.97)
Pancreas	8	1,217,983	0.98 (0.95–1.007)
Liver	6	911,858	0.94 (0.93–0.96)
Colorectal	16	2,234,441	0.98 (0.97–0.99)
Bladder	5	641,336	0.96 (0.92–0.99)
Prostate	9	524,358	0.99 (0.98–1.02)
Breast	23	911,884	0.95 (0.92–0.98)
Ovarian	2	233,591	1.0 (0.96–1.06)
Endometrial	3	6633	0.91 (0.80–1.03)
Melanoma	3	68,936	0.94 (0.87–1.01)
All sites	8	871,748	0.98 (0.96–1.002)

CI, confidence interval

Adapted from the Italian Institute of Health’s guidelines on the Mediterranean Diet [30]

Although no significant relationship was reported for certain cancer localizations, results may be driven by methodological factors. For example, for Pancreatic cancer, the level of evidence was judged to be low, despite the inclusion of over 1.2 million individuals in the analysis. Indeed, a recent meta-analysis that included 8 studies and totalled 1,301,320 subjects, showed that higher adherence to the Mediterranean diet was associated with a significant reduction in the risk of pancreatic cancer (hazard ratio 0.82 (0.76–0.88), *p* < 0.001) [31]. Notably, this meta-analysis examined the effect of high versus low adherence to the diet on cancer risk, whereas the analysis in the guidelines gave the relative risk associated with a 1-point increase in adherence, which may be a difficult metric to operationalize or measure accurately. Differences in the populations included or in the duration of follow-up, the low number of studies or their low methodological quality could also explain the lack of observed benefit of the Mediterranean diet on other cancer types.

In terms of overall cancer mortality, analysis of a total of 15 cohort or case-control studies, totalling 1,821,423 participants, and 31,327 events over follow-up ranging from 5 to 26 years, found that a 1-point increase in adherence to the Mediterranean diet was associated with a significant reduction in the risk of mortality (relative risk 0.97, 95% confidence interval (CI) 0.96–0.99) [30].

These findings have important implications for public health. Promoting adherence to the Mediterranean Diet can be a cost-effective strategy for cancer prevention, as there is abundant epidemiological evidence in favor of a beneficial effect of this dietary pattern on human health. To this end, nutrition education and public health policies should encourage whole foods, plant-based meals, and healthy fats (e.g., extra virgin olive oil). Furthermore, integrating dietary recommendations into clinical practice could enhance preventive oncology strategies.

Regarding Cantonese cuisine, several factors influence the relationship between the Cantonese diet and cancer incidence. First and foremost, cooking methods matter, for example with potential for causative links arising from nitrates and nitrosamines in preserved or processed dishes; or formation of polycyclic aromatic hydrocarbons from high-heat cooking. Double-boiling is a popular technique, but while prolonged cooking may better preserve nutrients, it also concentrates heavy metals. It also concentrates purines, increasing gout and metabolic risks. Similarly, claypot rice, another popular cooking technique in Cantonese cuisine, may result in charred crust laden with acrylamide. There is also some research about the association of Cantonese cuisine with lower cancer morbidity [32–34]. In this regard, Lin.et al. reported that a non-medicinal herbal diet was inversely associated with nasopharyngeal carcinoma [35]. If replicated, this result could hold potential for nasopharyngeal carcinoma prevention in endemic areas, such as Guangdong province. This is very interesting and it might give a new insight and study strategy in this field. Protective elements also abound in the region’s diet. Traditional Kongfu tea contains bioactive molecules linked to anticancer activity, such as polyphenols (which scavenge free radicals and inhibit tumor cell proliferation), theaflavins (which enhance antioxidant defenses and modulate inflammatory pathways), as well as vitamins and minerals that support immune function and DNA repair, and activate detoxifying enzymes. In addition, the Kongfu tea practice is in harmony with cancer-preventive lifestyle principles, such as mindfulness to reduce stress and lower chronic inflammation, and social connection to support mental well-being. Minimally processed seafood (e.g., scallops, prawns), another key feature of Cantonese cuisine, is low in saturated fat but rich in selenium and zinc. Furthermore, Cantonese cuisine typically limits red meat intake, aligning with WHO guidelines that associate high red meat consumption with colorectal cancer risk.

## Functional foods and innovation

### Fruit and vegetables

Increased consumption of fruit and vegetables is desirable within the context of ensuring a better general dietary pattern. There is a general consensus among the international community that a Daily per capita intake of 400 g of a variety of fruits and vegetables yields beneficial effects at a population level. The role of fruit and vegetables in the diet is equally relevant, whether for preventing micronutrient deficiency (i.e. malnutrition-related) or non-communicable diseases (i.e. over-nutrition-related). It should be underlined that the benefits of fruit and vegetable consumption are reaped when they are consumed in conjunction with the other elements of the Mediterranean diet. Furthermore, the recommended Daily intake of 400 g corresponds to an annual average availability of 150 kg/capita, but averages for per capita supply may mask wide variations among regions and countries. For perishable fruits and vegetables, average supply figures need to be corrected for realistic post-harvest loss along the supply chain and for wastage. Indeed, fruit and vegetables rank highly among wasted food in the world, because they are fresh, hard to store and rapidly perished. In reality, after accounting for estimated post-harvest loss and wastage, there is a shortfall at a global level between actual availability of fruit and vegetables, and total needs, based on recommended intake goals at population level; and this, despite encouraging increases in world fruit and vegetable supply over the last 20 years.

There are several key reasons why fruit and vegetables are so important from a nutritional point of view. Briefly, they are low in calories, high in fiber and water, low in fat, and rich in vitamins and minerals. However, despite the established benefits, consumption of fruit and vegetables is consistently below target levels in many countries [11]. Women seem to have better intake than men, overall [11]. This suboptimal intake has direct repercussions on Health, and data from the Global Burden of Disease 2017 Diet Collaborators shows that a large proportion of mortality is attributable to dietary factors [11]. Moreover, it has long been established that higher consumption of fruit and vegetables is significantly associated with a lower risk of death [36]. Of note, the benefits increase up to 5 portions a day, and then plateau, with no further mortality reduction to be gained from any incremental consumption beyond that point [36]. Key studies also showed that a diet rich in fruit and vegetables could significantly improve systolic blood pressure [37, 38]. Since even a small reduction in blood pressure at population level leads to a reduction in stroke, it is unsurprising that a significant reduction in stroke has also been reported with vegetarian dietary patterns [39]. Fruit and vegetable consumption is also associated with a significant reduction in the risk of type 2 diabetes mellitus [40]. Data from the French large-scale NutriNet-Santé prospective cohort (2009–2019) showed that the reduction in diabetes was driven by all the various forms of dietary fiber found in fruit and vegetables (total, soluble, insoluble), and additionally, high consumption of fruit and vegetables was also associated with significant reductions in several other chronic diseases, and mortality, in that study [41]. Nevertheless, the authors underlined that the individual nutrients should not be considered separately, but rather, it is the overall dietary pattern as a whole that yields the benefit, and not necessarily each individual component. An umbrella review including 18 meta-analyses, encompassing a total of 298 prospective observational studies and 21 outcomes, reported highly significant summary results for CVD and CVD mortality, coronary artery disease, pancreatic cancer, and gastric cancer [42]. Furthermore, there was convincing evidence for a benefit of dietary fiber on pancreatic cancer, CVD mortality, and all-cause mortality, although only CVD and all-cause mortality were based on prospective studies [42].

Overall, the evidence clearly supports a convincing effect of fruit and vegetable consumption on several diseases. The various components of fruit and vegetables that may at least partially explain their effects are listed in Table 3. A varied diet will bring the consumer all the benefits, but boosting consumption of fruit and vegetables worldwide remains a constant challenge. Two key determinants are nutrition education, to raise public awareness about the importance of eating fruit and vegetables, and secondly, a competent and efficient supply chain. Areas of emphasis that could be amenable to intervention include the return of the home garden, urban/peri-urban horticulture and commercial production, with the need for an equilibrium between these three different supply chains.

**Table 3:** Components of fruit and vegetables, sources, and possible mechanisms mediating their beneficial effects

Phytochemical	Plant Source	Possible benefits
Carotenoids (beta-carotene, lycopene, lutein, zeaxanthin)	Red, orange and green fruits and vegetables	May inhibit cancer cell growth, improve immune response and work as antioxidants
Flavonoids (anthocyanins and quercetin)	Apples, citrus, onions, soybeans, coffee, tea	May inhibit inflammation and tumor growth, aid immunity and boost detoxifying enzyme production
Indoles and Glucosinolates (sulforaphane)	Cruciferous vegetables	May induce detox of carcinogens, limit cancer-related hormone production, block carcinogens, prevent tumor growth
Inositol (phytic acid)	Bran from corn, oats, rice, rye and wheat, nuts, soybeans	May slow cell growth and work as antioxidant
Isoflavones (daidzein and genistein)	Soybeans and soy products	May inhibit tumor growth, cancer related hormone production and work as an antioxidant
Isothiocyanates	Cruciferous vegetables	May detox carcinogens, block tumor growth and work as antioxidants
Polyphenols (ellagic acid and resveratrol)	Green tea, grapes, wine, berries, citrus fruit, apples, whole grains and peanuts	May prevent cancer formation, prevent inflammation and work as an antioxidant
Terpenes (perilly alcohol, limonene, carnosol)	Cherries, citrus fruit peel, rosemary	May protect cells from becoming cancerous, slow cell growth, strength immune system, fight viruses, antioxidant

In summary, fruit and vegetable consumption, with a recommended Daily intake of 400 g per person, is a cornerstone of healthy dietary patterns and supports overall health. Established health benefits include reductions in the risk of non-communicable diseases (such as CVD, stroke, hypertension or diabetes), and reduction mortality. However, consumption is below target in many countries, and gaps in availability remain apparent, with post-harvest losses and wastage reducing actual intake. Boosting consumption worldwide requires a focus on nutrition education and an efficient supply chain, with partnerships and strategic alliances essential for an integrated approach.

### Whole grains

Archaelogical findings show that grains have been grown and eaten for over 6000 years in Europe, and for over 12,000 years in Mesopotamia. After nomads and hunter-gatherers, the “neolithic revolution” switched to cultivation of cereals, as humans became sedentary. Historically, grains were always consumed whole, and the shift to refined grains happened only around 200 years ago, with the advent of new processing methods making it possible to obtain a finer texture, longer shelf-life and improved taste [43].

Whole grain is grain that retains the bran and the germ together with the endosperm [44]. The bran and germ layers are typically removed during the milling process to produce refined grains, which have a longer shelf-life, but have lost much of the nutrient goodness. In contrast, by retaining those parts, whole grain offers a great source of fiber and other important nutrients, such as B vitamins, iron, selenium, potassium, magnesium and phytonutrients [44]. Comparison of the nutritional value of whole versus refined wheat flour shows that the fiber content is three times higher and minerals (especially magnesium) much more abundant in whole grain flour, compared to refined flour [45]. The same applies for rice, with whole grain rice having higher content of important nutrients compared to its more refined counterpart [45].

There is strong and consistent evidence that whole grains play a major role in healthy and sustainable dietary patterns [46], and this report recommends a Daily consumption of 232 g of wholegrain for Health and sustainability of the food systems. Furthermore, there is increasing evidence to suggest that the consumption of whole grain can Help reduce the risk of non-communicable diseases including type 2 diabetes, cardiovascular disease, certain types of cancer and weight management [13, 47, 48].

As previously mentioned, dietary risks are the single greatest contributor to mortality, according to the Global Burden of Disease Study 2017. A diet low in whole grain is the second biggest risk factor in diet-related mortality rates, after a high-salt diet, and the leading factor in terms of years lost to disability [11]. There is a dose-response relationship between whole grain intake and cardiovascular outcomes based on data from prospective studies, with a threshold of about 50 g shown to yield optimal benefits [47, 48]. A diet rich in whole grains is also a protective factor for cancer mortality and total mortality [48]. Accordingly, this relatively minor whole grain intake of around 50 g per day could lead to substantial economic benefits by reducing Healthcare expenses and minimizing lost productivity. Quantitative recommendations indicate that 50 g is around 3 portions per day, with a portion comprising 16 g of dry weight [49]. The potential savings in terms of healthcare are immense, as testified by an analysis of four economic studies that evaluated the healthcare cost savings impact of increasing whole grain intake. The results in terms of savings were similar and significant for the US, Australia, and Finland for the various chronic diseases studied [50].

Yet despite the recognized Health and economic benefits, consumption remains low. At world level, most countries have mean consumption levels below 15 g per day, well below the target 50 g [51]. In China and in Italy and most Mediterranean countries, whole grain consumption was less than 15 g per day in 2010 [52]. A more recent study from nutrition surveillance for the year 2015 indicated an average consumption of 19.8 per day, in China, where more than 80% of adults severely under-consume whole grains [53].

Although food-based dietary guidelines recommend whole grain consumption, there is no consensual, firm recommendation about quantities. Various examples can be found, ranging from 48 g per day recommended in the USA, to 75 g recommended in Nordic countries and up to 150 g in China. Yet, although consumers need concrete guidance, since the appropriate number of portions will depend on the whole grain content of the product. This requires a fixed definition of a “whole grain food”, but to date, a global consensus is lacking. In this regard, the Whole Grain Initiative, a not-for-profit based in Switzerland, has been actively supporting efforts to harmonize practices, notably by striving to create an International Standards Organisation (ISO) standard for whole grain foods, to be published imminently, and should lead the way for government-led initiatives and industry standards. The Whole Grain Initiative is also aiming to publish a CODEX standard that can be used by authorities in the absence of a local definition. A further step is the clear identification of whole grain foods, by incorporating it into front-of-pack labels, e.g. through public-private partnerships. Initiatives of this type have been implemented in Nordic countries and achieved a substantial increase in consumption [54]. Importantly, definitions for whole grain ingredients and foods have been published by a panel of independent scientific experts, stipulating that “A whole-grain food shall contain at least 50% wholegrain ingredients based on dry weight; and foods containing 25–50% whole-grain ingredients based on dry weight, may make a front-of-pack claim on the presence of whole grain but cannot be designated ‘Whole Grain’ in the product name” [55]. The establishment of a consensual definition of “whole grain” foods as well as labelling criteria will pave the way for the next steps, such as recommendations for dietary intake at national level, the inclusion of whole grain criteria in front-of-pack nutritional labelling schemes (e.g. Nutriscore in Europe, HSR in Australia, Healthy Choice China). In this regard, the Chinese government actively promotes the whole grain industry and increasing whole grain consumption, with the use of dedicated and easily identifiable “Whole Grain” stamps.

Overall, there is an abundant body of evidence attesting to the health and economic benefits to be yielded from increased consumption of whole grains. Several quantitative recommendations exist for optimal levels of whole grain consumption. However, consumption remains low overall, and efforts to promote increased intake are hampered by the absence of a consensual international definition of “whole grain” foods or ingredients. Initiatives by organisations such as the Whole Grain Initiative should help to achieve harmonization of definitions, recommendations and practices in the coming years, for the benefit of human health.

### Fermented dairy products

Milk-based products have been around for at least 6 millennia, as evidenced by the presence of dairy proteins found in human dental calculus from northeastern Africa [56]. There is also evidence of the use of fermentation to preserve food, and render it less perishable and thus more transportable, as well as more digestible (since there is less lactose content after fermentation) [57].

Fermented dairy products have demonstrated positive effects on fracture, CVD, diabetes mellitus, cancer and neurodegenerative diseases.

The consumption of fermented dairy products is associated with attenuated cortical bone loss, independently of total calcium, protein, and energy intake, in healthy postmenopausal women, with bone loss markedly attenuated in women who take at least one serving per day [58]. There is abundant evidence supporting the fact that the consumption of fermented dairy products, such as yoghurt or cheese, is associated with a reduced risk of fracture [59–61]. The mechanisms of this relation are complex, and are mediated via calcium, pre/probiotics and protein, to ultimately increase bone mineralization and formation, and reduce bone resorption [62].

Regarding CVD, an umbrella review and meta-analysis of prospective studies found a significant reduction in overall CVD, as well as in the risk of coronary heart disease, hypertension and stroke with increasing cheese consumption [61]. Similarly, another meta-analysis combining data from 29 prospective cohort studies reported that increased intake of total fermented dairy (including sour milk products, cheese or yoghurt; per 20 g/day increment) was associated with a significantly reduced risk of all-cause mortality (relative risk (RR) 0.98, 95% CI 0.97–0.99) and CVD risk (RR 0.98, 95% CI 0.97–0.99) [63]. It has been posited that the dairy matrix (i.e. physio-chemical structure) is more than the sum of the individual parts, and it is the form in which fermented dairy products are consumed that is most important [64]. Indeed, in a randomized trial, Feeney et al. tested the impact of Daily consumption over a period of 6 weeks of 40 g of dairy fat on markers of metabolic health in overweight adults [65]. Interestingly, the authors observed that the group that consumed the dairy fat solely contained within cheese had decreased LDL, but no such effect was seen in those who consumed the same ingredients given as butter/salts (i.e. within a different nutrient matrix). This underlines the importance of considering the overall dietary pattern, rather than individual nutrients in research and in guidelines for nutrition.

Both yoghurt (high-fat only) and cheese have also been shown to be associated with a lower risk of prediabetes in two studies from the Netherlands [66, 67]. In both studies, the high-fat fermented products were inversely associated with prediabetes risk, despite the higher fat content. Similarly, fasting plasma glucose, glycated hemoglobin (HbA1c), total cholesterol and C-reactive protein were all found to be lower with increasing intake of fermented dairy products [68]. A meta-analysis bringing together 15 studies, and including a total of 485,992 participants and 20,207 incidences of diabetes showed that overall, there was a significantly reduced risk of diabetes with higher intake of fermented dairy foods (odds ratio [OR], 0.925; 95% CI, 0.856–0.999). Specifically, higher yoghurt consumption was significantly associated with a decreased diabetes risk (OR, 0.828; 95% CI, 0.729–0.941). Finally, several key parameters of the cardiometabolic syndrome (e.g. hyperglycemia, hypertension, hyperlipidemia, and possibly overweight), also appear to be beneficially influenced by higher yoghurt consumption [69].

The effect of fermented Dairy products on neurodegenerative diseases is less well established. In a cross-sectional analysis performed on baseline data from 6744 adults (aged 55 to 75 years old) in the Predimed cohort, Munoz-Garach et al. reported lower Mini Mental State Examination (MMSE) scores in subjects with higher intake of fermented dairy products [70]. However, findings from a two-year study from the Predimed-Plus cohort showed no clear associations between intake of the most commonly consumed dairy products (most milks, cheese, yoghurt) and cognitive performance in this European study [71]. One longitudinal study from Japan that analyzed the impact of dairy intake on incident Functional disability in almost 12,000 adults aged over 65, followed for an average of over 8 years, showed a trend towards reduced risk of dementia in consumers of yoghurt, a trend towards an increased risk in cheese consumers, but the associations were no longer significant after adjustment for education, smoking and alcohol status, as well as physical and psychological factors including risk factors and history of disease [72]. A study from the Netherlands corroborated the positive impact of cheese consumption on cognitive Function, reporting that a 30 g increase in Dutch cheese intake was associated with significantly better information processing speed; however, no associations were observed between dairy consumption and other aspects of cognitive function, such as attention, working memory or episodic memory [73]. A randomized study using Functional magnetic resonance imaging reported that 4 weeks of intake of fermented, probiotic-containing milk products by healthy women affected activity of the brain regions that control central processing of emotion and sensation, providing insights into the possible mechanisms of action of fermented dairy products in improving cognitive function [74].

Finally, the literature is less convincing regarding the impact of fermented dairy products on the risk of cancer. Higher yoghurt consumption may be associated with colorectal cancer, but the evidence is uncertain [75]. Results from meta-analyses examining the association between dairy product intake and prostate or bladder cancer are inconclusive [76, 77], and a meta-analysis combining 184 observational studies showed no significant association of cheese consumption with cancer of any type [61].

To conclude, meta-analyses of both observational studies and randomized clinical trials report no harmful effects of Dairy products on body fat, metabolic syndrome, type 2 diabetes, or CVD. On the contrary, meta-analyses of observational studies actually support a possible protective effect of full-fat yoghurt and cheese (and perhaps other fermented dairy products) against CVD, type 2 diabetes, and osteoporotic fractures. The effects of yoghurt and cheese on body composition, diabetes and CVD risks can likely be attributed to the food matrix. The exact mechanisms behind these benefits remain unclear, and may be due to the fact that consumption of fermented dairy products is a marker of more healthy behaviours. For example, yoghurt consumption has been hailed as a hallmark of a healthy lifestyle, whereby people who eat yoghurt eat more healthily overall, exercise more and smoke less, and the carbon footprint of yoghurt production is lower than other foods [78]. However, this does not exclude the possibility that there are also direct Health benefits, and thus, a diet including Dairy products, particularly fermented products such as yoghurt and cheese, should be recommended to prevent type 2 diabetes, cardiovascular disease and osteoporosis.

### Extra virgin olive oil

Extra virgin olive oil (EVOO) is a key component of the MedD, with powerful antioxidant and anti-inflammatory properties. The major fatty acids in olive oil triacylglycerols are oleic acid, linoleic acid, palmitic acid, stearic acid and linolenic acid, while the free fatty acid content of EVOO should be < 0.8%. EVOO is unrefined, in contrast to regular olive oil, or oils from other sources, which may lose many of their beneficial components through refining. The composition of olive oil depends on the cultivar, climate, maturity of the fruit, processing and storage. Colder climates tend to yield oil with a higher oleic acid content than warmer climates, although this depends on the cultivar and region. A higher content in monounsaturated compared to polyunsaturated fatty acids renders the olive oil more resistant to oxidation because the more bonds there are, the more easily they can be broken down by phenomena such as heat, light or oxidative stress. EVOO also contains essential micronutrients, notably vitamin E, a natural antioxidant, and vitamin K, with olive oil being the second best source of vitamin K, after leafy green vegetables.

There are many polyphenols in EVOO, and these are the most bioactive substances, contributing to the protection of blood lipids from oxidative stress, for example. The minimum polyphenol content to qualify for the denomination “extra virgin” olive oil is 250 parts per million. It is essential to preserve the chemical composition of EVOO in order to protect its beneficial properties. This includes controlling factors such as storage, temperature, light and oxygen exposure [79].

Several lines of evidence provide evidence in support of the beneficial effects of EVOO within the context of a Mediterranean dietary pattern [80]. These include cardioprotective effects [81, 82], and benefits in the management of metabolic disorders [83], particularly type 2 diabetes [84]. In addition, a diet enriched in EVOO has been shown to have protective effects on bone health [85, 86].

The use of raw EVOO added to foods after cooking (or as a salad oil) is the best way to express the original flavour and to maximize the intake of natural anti oxidants and EVOO compounds associated with positive effects on human health. However, EVOO also exhibits protective properties during cooking, whereby chemical interactions between the oil’s biophenolic compounds and the other ingredients may increase in the overall antioxidant properties.

Overall, consuming EVOO as an integral part of a healthy diet is a preventive measure that may at least partially mitigate or slow down aging and inflammation.

### Herbs and herbal infusions

The MedD is widely recognized for its health-promoting properties and association with increased longevity. While its core components—olive oil, vegetables, legumes, and whole grains—are well-documented, herbs and herbal infusions represent a critical yet underexplored dimension. Traditionally used across the Mediterranean and globally for culinary, aromatic, and therapeutic purposes, herbs enhance not only flavor, aroma, and color but also serve as substitutes for salt [87]. Their contribution to nutritional density stems from their richness in bioactive compounds, including flavonoids, vitamins, and minerals.

Herbal infusions, distinct from caffeinated teas, are a fundamental part of the traditional MedD and recommended as water alternatives throughout the region. The European Medicines Agency (EMA) classifies their preparation as either infusions (hot water poured over herbs) or decoctions (cold water brought to boil with herbs). Archaeological findings, such as 3,500-year-old Cretan strainers, underscore the long-standing cultural significance of these practices. Crete, in particular, hosts numerous endemic herbs—e.g., *Sideritis* (mountain tea), dittany, marjoram, and sage—valued for both culinary and medicinal properties. Historical accounts link *Sideritis* (locally termed malotira) to health benefits, a notion partially supported by modern pharmacological recognition for respiratory and gastrointestinal relief.

Despite their cultural and therapeutic relevance, endemic herbs face ecological threats due to overharvesting, urban development, and climate change. High market demand has prompted sustainable cultivation initiatives to counter unsustainable wild collection. Climate variability also affects the phytochemical composition of herbs, which produce polyphenols and antioxidants as adaptive responses to environmental stressors. These biochemical shifts may influence both therapeutic efficacy and dietary value, necessitating further study.

Food preparation methods play a vital role in modulating the nutritional impact of herbs and oils. In Greek cuisine, *tsigarisma*—a technique akin to Spanish sofrito—involves gently warming olive oil with herbs, garlic, and vegetables. This method extracts phytochemicals into the oil without degrading volatile compounds, enhancing both flavor and health potential. Data from the HYDRIA nutrition survey in Greece indicates that herbs like dill, parsley, oregano, and mint are widely consumed, especially among older adults [88]. Herbal teas remain popular, though their consumption has declined among younger generations, who increasingly favor green and black teas.

Epidemiological evidence supports the MedD’s protective role against chronic diseases, but mechanistic insights remain incomplete. Ongoing research led by Trichopoulou et al. seeks to identify polyphenol metabolites in blood samples, beginning with *Sideritis* [89]. Their findings may elucidate pathways linking herbal infusions to health outcomes.

Finally, the Mediterranean Basin, as a global biodiversity hotspot, holds significant promise for the sustainable use of medicinal and aromatic plants (MAPs). Yet, this promise is tempered by risks such as mycotoxin contamination—exacerbated by inadequate storage or pesticide use—highlighting the need for balanced safety regulations. Continued interdisciplinary research, cultural preservation, and policy support are essential to integrate these herbal traditions into modern preventive nutrition strategies.

The use of herbal tea is also regarded as a popular primary healthcare system in many countries especially in China [90]. In the Cantonese diet, Cantonese herbal tea (CHT, known as Liangcha in Mandarin and Leungcha in Cantonese) is a very important element, originating over 2,000 years ago, during the Qin and Han dynasties (221 BCE–220 CE). According to the index of intangible cultural heritage, CHT is an everyday drink made from Chinese herbs, based on the distinctive local weather and the water/soil quality, by people from Guangdong, Hong Kong and Macao. These populations developed the drinking habit from their long-term experience of disease prevention and healthcare with particular guidance from Chinese medicinal theories of health to dispel “heatiness” and detoxify, as well as to prevent infection [91]. The hot, humid summers in southern China’s Lingnan region led to widespread issues like “heat” or “heatiness” inside human bodies (internal heat, called Shanghuo in Mandarin and Yeehay in Cantonese), which were thought to cause illnesses, such as sore throat, mouth ulcers, constipation, acne, bad breath, headaches, dizziness, fever and cough. This “heatiness” describes a status of Yin-Yang imbalance when Yang overwhelms Yin. The imbalance of Yin-Yang resembles the breaking of homeostasis and manifests by impaired physiological functions, which leads to the onset, recurrence, and progression of many diseases [92]. CHT recipes typically incorporate herbs that can remove heat and detoxification, such as Prunella vulgaris (Xia Ku Cao) and Lonicera japonica (Jin Yin Hua) [93]. The herbs in CHT contain flavonoids, organic acids, alkaloids, polysaccharides and glycosides, etc., which supply CHT with anti-oxidatant, anti-inflammatory, hypoglycemic, antiviral, antibacterial, anti-tumor, anti-aging and hepatoprotective effects [94], relieving uncomfortable symptoms and helping to prevent serious diseases. There are studies suggesting that CHT or the ingredients of herbal tea correlate with treating diseases like upper respiratory infections [95].

In contemporary China, CHT has transitioned from household preparations to industrialized production. Future development hinges on standardizing cultivation practices, validating health claims through randomized trials, and innovating palatable formats (e.g., effervescent tablets or functional snacks). Integrating TCM wisdom with evidence-based nutrition could position CHT as a global functional food, provided regulatory frameworks address safety and sustainability concerns.

### Research & innovation

The Mediterranean dietary pattern has been recognized by UNESCO as an Intangible Cultural Heritage of Humanity due to its documented health benefits and sustainable food practices, shown to be associated with longevity, lower risks of non communicable diseases, and overall well-being.

Nevertheless, even with the framework of the MedD, there is potential for nutrient deficiencies, for example due to low intake of micronutrients (e.g. vitamins, minerals), or an imbalanced ratio of omega-3 to omega-6 fatty acids. Low bioavailability of beneficial compounds within the elements of the MedD foods could lessen the overall benefits to be yielded through consumption. There is also some loss of nutritional quality during food processing, notably with degradation of vitamins, polyphenols and antioxidants.

These potential pitfalls can be circumvented by calling on scientific solutions being developed currently by the Nutrage project, a multidisciplinary initiative in Italy, coordinated by the National Research Council (CNR), with the main aim of promoting active and Healthy aging through innovative research in nutrition, biotechnology, and personalized Health strategies. In this research programme, 36 different institutes from 5 CNR departments are collaborating in areas such as food and agriculture (bio)technologies, development of novel foods with high health value, new technological solutions for transforming foods, and innovative strategies for active and healthy aging.

Some of the solutions being studied include biofortification of microgreens, the young and tender sprouts of vegetables, herbs, legumes or cereal seeds, which can be fortified in vitamins or minerals without changing the production parameters or the commercial quality of the product. Fermentation is also being explored for its ability to improve the functional properties of pulses’ flour (e.g. water absorption index, water holding capacity, fat absorption and emulsifying properties), and improve their nutritional value in terms of total dietary fiber, protein digestibility, mineral, amino acid, fat and antioxidant contents of composite breads. Fermentation also enhances physical properties (such as specific volume and crumb firmness), improving appearance, texture, color and overall acceptance. These advancements aim to enhance nutrient density and functionality without sacrificing cultural integrity. Clinical studies support the use of high-amylose grains and fermented vegetables for improved glycemic control and lipid profiles. These novel technologies can be a useful tool to further improve the nutritional value of major and minor crops, but the (re-) discovery of niche crops, local varieties and herbs is also strategic to achieve the nutritional goals of the MedD. Above all, societal engagement is fundamental for boosting the strategic importance of MedD in the context of a “One Health” concept.

### Food-medicine homology

Food-medicine homology refers to the concept that certain foods have both nutritional and medicinal properties, and there is no strict boundary between food and medicine. In Chinese medicine theory, many substances can be used not only as daily food to provide energy and nutrients, but also as medicines to prevent and treat diseases and maintain health. In the Cantonese diet, dietary substances are classified according to their thermal (hot/cold) and flavor (sweet, sour, bitter, spicy, salty) properties, supporting a personalized approach to preventive nutrition. The Chinese Health authorities have released four catalogues with 106 substances that serve a dual purpose as both food and traditional Chinese medicinal substances. In this paradigm, foods are selected based on their ability to regulate bodily functions in accordance with traditional Chinese medicine principles.

There are several examples of common substances with attractive properties for both food and therapeutic uses. One such example is Chenpi (dried tangerine peel), widely used as a food, but whose bioactive properties have also shown promise in clinical applications for human health [96–98]. With the advancement of technology, the manufacturing of modern Chenpi is increasingly trending towards scientific, automated, and intelligent processes [99]. Another example is cinnamon, with an umbrella review reporting that cinnamon supplements can significantly improve systolic and diastolic blood pressure, enhance total antioxidant capacity, and reduce IL-6 levels [100]. More recent research suggests that the addition of cinnamon to the diet promotes longevity, an effect that is mediated through mTORC1 and autophagy signaling pathways [101].

In summary, food-medicine homology promotes health-focused dietary practices by prioritizing seasonal and locally sourced foods, aligned with natural laws (e.g., traditional Chinese medicine’s “harmony between humans and nature”). The ingredients maximize both the nutritional and functional benefits [102]. Modern food-medicine homology principles are underpinned by a growing body of scientific evidence, while remaining mindful of sustainable development. Interdisciplinary cooperation and intelligent application will help transform these principles into practical strategies that enhance public health, reduce disease burden, and promote ecological resilience.

## Public health and policy considerations

From a public health and policy perspective, there are a number of key considerations. Malnutrition in all its forms—undernutrition, obesity, and micronutrient deficiencies—remains a global crisis, with unhealthy diets at its core. Despite extensive evidence supporting the health and environmental benefits of the MedD, modern food systems and policy environments fail to support its widespread adoption. Current systems prioritize ultra-processed foods and lack infrastructure for sustainable, nutritious food production and access. Public health strategies must go beyond individual dietary choices to address systemic barriers such as food marketing (particularly to children), time constraints, cost perceptions, and limited cooking skills. Fiscal policies like value added tax (VAT) reductions are insufficient alone; instead, a coordinated, multi-sectoral approach is needed, spanning health, education, agriculture, and social protection, to increase equitable access to the MedD. Effective promotion also requires better regulation, increased health promotion funding, and culturally relevant public education to drive lasting dietary change.

Stakeholders from public health, science, and civil society emphasized the role of healthy dietary patterns, such as the MedD and Cantonese cuisine, in improving health, sustainability, and social well-being. They highlighted the urgent need for better education, stronger food policies, and integration of MedD principles into public systems. Challenges such as low adherence, marketing pressures, and systemic policy gaps were widely acknowledged. Collaborative platforms like Sabir and Salus were presented as tools to bridge science, culture, and practice. The Sabir initiative is a knowledge-sharing platform developed by the Food and Agriculture Organization as an observatory to promote the MedD through integrated insights on nutrition, environment, and community. The European Salus network, coordinated by Lumen, a non-profit organization in Italy, advocates for health promotion policies and lifestyle interventions, including the MedD, across multiple sectors. A list of concrete Calls to Action by the participating stakeholders are outlined in Table 4, summarizing key recommended initiatives.

**Table 4:** Call to action by stakeholders: promoting the mediterranean diet

1. Develop and Implement Cross-Sector Policies • Integrate MedD principles into health, education, agriculture, and social welfare policies. • Increase budget allocations for health promotion beyond traditional healthcare spending.
2. Create a Central European Hub for Health Promotion • Establish a European Centre for Health Promotion with a focus on lifestyle interventions like the MedD.
3. Support Nutrition-Sensitive Food Systems • Reform food procurement in public institutions (schools, hospitals) to include MedD-aligned foods. • Promote sustainable agricultural practices that support MedD components.
4. Strengthen Education and Training • Develop standard qualifications for health promoters who can educate the public about MedD and behavior change. • Include MedD education in school curricula and health professional training.
5. Improve Public Awareness and Accessibility • Launch public campaigns using clear, practical messages about the benefits and application of the MedD. • Address social and economic barriers, such as time, cost, and cooking skills.
6. Enhance Food Labelling and Consumer Information • Include whole grains and MedD-aligned features in food labelling systems (e.g., front-of-pack indicators). • Regulate food marketing, especially to children, across digital platforms.
7. Leverage Digital and Community Platforms • Support platforms like Sabir to share scientific, environmental, and cultural knowledge about the MedD. • Translate research into accessible content for different audiences.
8. Foster International Collaboration • Promote trilateral meetings among Europe, India, and China on traditional medicine and dietary heritage. • Exchange knowledge and best practices with global partners to enrich MedD implementation.
9.Promote Research and Innovation • Fund interdisciplinary research into new MedD-related interventions, including functional ingredients (e.g., mushrooms). • Investigate the environmental and health benefits of underutilized MedD components.
10. Monitor and Evaluate Policy Impact • Track adherence to the MedD at the population level. • Assess the impact of promotional policies and campaigns through robust public health metrics.

MedD, Mediterreanean diet

## Conclusion

The Mediterranean and Cantonese dietary systems are more than nutritional models; they are cultural, ecological, and medical paradigms. Both exemplify how traditional knowledge can align with scientific evidence to promote lifelong health, offering protective effects against cardiovascular disease, cancer, and metabolic disorders. Their emphasis on diverse plant-based ingredients, functional foods, and mindful culinary traditions reflects a holistic approach that transcends simple nutrient metrics. However, realizing their full potential, and incorporating these traditions into contemporary dietary recommendations requires not only scientific validation through research, but also systemic support through integrated policy, public education, and culinary and technological innovations that make these traditions accessible and relevant to modern lifestyles.

As global health systems struggle with the dual burden of obesity and malnutrition, heritage dietary patterns like the Mediterranean and Cantonese diets, offer sustainable, culturally resonant solutions. Yet, their widespread adoption hinges on overcoming barriers such as food marketing pressures, convenience-driven eating habits, and lack of infrastructure for healthy food environments. Future research should focus on the synergistic effects of food combinations, longitudinal outcomes of dietary interventions, and effective strategies for cultural preservation and modernization. Multi-stakeholder collaboration, exemplified by initiatives such as this bilateral meeting, is essential to bridge science and culture, ensuring that by revitalizing and adapting traditional dietary wisdom, we can foster a healthier, more resilient global population grounded in the culinary and cultural richness of our past.

## Data Availability

No datasets were generated or analysed during the current study.
